# Fast and Powerful: Biomechanics and Bite Forces of the Mandibles in the American Cockroach *Periplaneta americana*

**DOI:** 10.1371/journal.pone.0141226

**Published:** 2015-11-11

**Authors:** Tom Weihmann, Lars Reinhardt, Kevin Weißing, Tobias Siebert, Benjamin Wipfler

**Affiliations:** 1 Dept. of Zoology, University of Cambridge, Cambridge, United Kingdom; 2 Science of Motion, Friedrich Schiller University Jena, Jena, Germany; 3 Entomology Group, Institut für Spezielle Zoologie und Evolutionsbiologie mit Phyletischem Museum, Friedrich-Schiller-Universität Jena, Jena, Germany; 4 Institute of Sport and Motion Science, University of Stuttgart, Stuttgart, Germany; University of Arizona, UNITED STATES

## Abstract

Knowing the functionality and capabilities of masticatory apparatuses is essential for the ecological classification of jawed organisms. Nevertheless insects, especially with their outstanding high species number providing an overwhelming morphological diversity, are notoriously underexplored with respect to maximum bite forces and their dependency on the mandible opening angles. Aiming for a general understanding of insect biting, we examined the generalist feeding cockroach *Periplaneta americana*, characterized by its primitive chewing mouth parts. We measured active isometric bite forces and passive forces caused by joint resistance over the entire mandibular range with a custom-built 2D force transducer. The opening angle of the mandibles was quantified by using a video system. With respect to the effective mechanical advantage of the mandibles and the cross-section areas, we calculated the forces exerted by the mandible closer muscles and the corresponding muscle stress values. Comparisons with the scarce data available revealed close similarities of the cockroaches’ mandible closer stress values (58 N/cm2) to that of smaller specialist carnivorous ground beetles, but strikingly higher values than in larger stag beetles. In contrast to available datasets our results imply the activity of faster and slower muscle fibres, with the latter becoming active only when the animals chew on tough material which requires repetitive, hard biting. Under such circumstances the coactivity of fast and slow fibres provides a force boost which is not available during short-term activities, since long latencies prevent a specific effective employment of the slow fibres in this case.

## Introduction

Feeding is one of the basic manifestations of life. In higher taxa, it is generally implemented by the action of specialized apparatuses, which are characterized in many vertebrates and arthropods by jaws that interact with each other. The significance of these ingestion instruments is shown by the considerable body of literature concerning their functional morphology, neuro-mechanics, control, as well as their ecological and behavioural impact (e.g. [1–5]). One of the most significant measures is maximum voluntary bite force. These forces are of vital importance specifically during chopping and grinding of tough food items, and during defensive behaviour and prey capture. Here they determine the maximum toughness of exploitable food or prey and whether opponents can be fended off by biting or if it is better to elope. Existing studies focussing on biting forces examine mainly vertebrates and to some extent larger arthropods like crustaceans. To our knowledge only two studies focus on biting forces in insects at all. One very recent study deals with bite forces of stag beetles but focuses on the typical marked sexual dimorphism, with males with enlarged and strongly modified mandibles [6]. Moreover, an older study focused on maximum bite forces in ground and rove beetles [7]. Except for the study regarding stag beetles, where the gape of the male mandibles was varied to some extent, gape variation has not been examined. Thus, for the largest group of terrestrial arthropods contributing a major part of the faunal biomass, reliable determinations of bite forces over the functional angular range are almost completely lacking, though studies regarding behaviour and energetics of feeding in ants and other durophagous species showed extraordinary high metabolic rates when cutting up hard food [8]. These results, in turn, suggest strong bite forces being a common characteristic also for insects.

**Fig 1:**
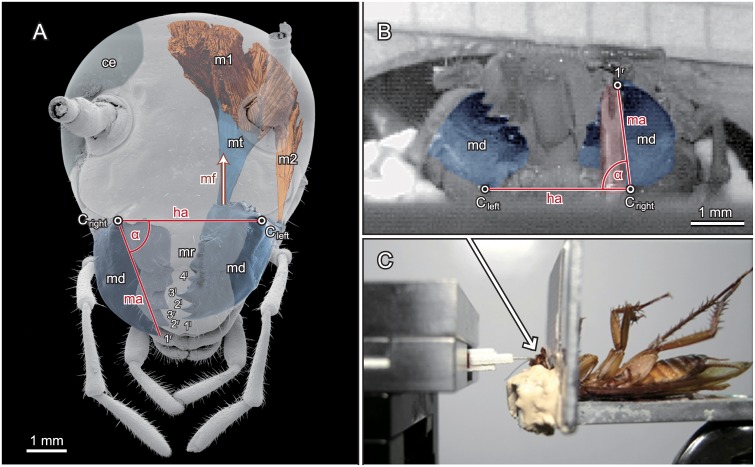
Morphology of the mandibular apparatus of *P*. *americana*, kinematics and general experimental setup. A) Morphology of the cockroach head from a μCT scan with emphasis to the mandibles (coloured light blue) and their driving muscles (light red). 1^l^-4^l^: teeth of the left mandible; 1^r^-3^r^: teeth of the right mandible; α: opening angle of the right mandible (approx.. 70°); ‘ce’: right complex eye; ‘md’: mandibles; ‘C_left_‘ and ‘C_right_’: anterior condyles of the left and right mandible joints; ‘m1’: left mandible closer muscle; ‘m2’: left mandible opener; mf: main direction of the muscle force; ‘mt’: apodeme connecting the mandible closer muscle with the median edge of the mandible; mr: molar region B) Camera view onto the mandibles (light blue) and the sensor tip (light red) during the bite experiments. The horizontal line is defined by the anterior condyles of the left and right mandible joints C_left_ and C_right_; ‘1^r^’ depicts the distal tip of the right mandible and α is the mandible angle with respect to the horizontal line, i.e. the opening angle of the right mandible before mathematical correction (see methods). C) Side view of the general setup with the fixated specimen at the right and the force transducer at the left side.

In insects with biting mouthparts a pair of slightly asymmetric bladelike structures, the mandibles are working against each other. They are the strongest mouthparts and play a major role in reducing larger food items in smaller digestible pieces. Distally they usually have a varying number of cuticular teeth (incisivi) that interdigitate when the mandibles are closed, while the meso-proximal side holds a grinding area, the molar region. Next to shredding food, mandibles are also regularly used for digging, feeding nest mates or offspring, clinging, transport, defence or other activities [9]. From a functional point of view, mandibles rely on relatively simple mechanisms, and can easily be understood as two class 3 levers working against each other [2] by transmitting forces generated by the closer muscles to teeth, edges or grinding ridges at the distal parts. In neopteran insects, i.e. in all flying insects with the exception of mayflies, dragonflies, and damselflies, the mandibles are connected to the head capsule via a simple hinge joint whose rotational axis is defined by an anterior and a posterior condyle [10–12]. Thus, they can move only in a single plane. Thereby a very limited set of two to five muscles is associated with the mandible [13]. However, the actual biting forces are generated almost solely by a single mandible closer muscle. Its antagonistic opener muscle is much smaller with only about 1/10 of the cross sectional area and has a much lower mechanical advantage [11,14]. Since teeth or cusps of both sides interlock when the mandibles close, in many insect species the left and right mandibles are slightly asymmetric [1]. In contrast to many herbivorous insects (cp. [2]) the mandible cutting edges of the omnivorous *Periplaneta americana* are not oriented in parallel to the joint axes but rather perpendicular like scissor blades (Fig 1). This way they resemble rather the configuration found in carnivorous insects [15]. Additionally, the molar region is small compared to phytophagous insects [14, 16]. Ingestion seems to be limited to small particles and soft matter. However, the second tooth of the right mandible (2^r^ in Fig 1) and the third tooth of the left (3^l^ in Fig 1) form a pair of sectorial teeth with sharp edges [14,17]. This pair seems, in turn, to facilitate cutting up stringy matter such as fibrous plant and animal materials.

In contrast to vertebrates, arthropods are characterized by exoskeletons, and therefore by muscles that are enclosed in rigid shells. As a consequence, the muscles are attached to the inner sides of the respective skeletal elements, and maximum shortening is determined not only by the margins that the specific joint structure permits [18,19] but also by the diameter of the skeletal element that encloses the muscle as it limits muscle thickening [10,20]. Typically insect muscles in confined cuticular spaces show an oblique fibre (pinnate) arrangement to reduce thickening when getting contracted. The pincers of scorpions, crayfish and crabs, and the chelicerae of solifugae as well as the mandibles of stag beetles can generate extraordinary strong forces with regard to body weight [6,21]. At least in crustaceans, the strong forces rely on muscle fibres with particularly long sarcomeres of up to 17 μm length [22], but also in the closer muscles of ants fibre bundles with relatively long sarcomeres (5–9 μm) were found [10,23]. In contrast to crustaceans and chelicerates, the strongest ‘biting’ forces of insects rely on mandibles whose driving muscles are found within the head capsule and not within peripheral appendages. Thus, muscles may compete for space with other essential organs, like the central nervous or the digestive system, which makes functional differences and specific characteristics conceivable.

In vertebrates, maximum muscle stress values are very similar for all striated skeletal muscles and always close to 30 N/cm^2^ [20,24]. In arthropods, however, maximum stress varies considerably even within a single specimen, depending on the major purpose of a certain muscle [18,25,26]. Their values range from about 8 N/cm^2^ in the legs of stick insects up to more than 100 N/cm^2^ in crayfish pincers [18,21,22]. Therefore, the increasing number of anatomy based studies providing morphological descriptions of the skeleto-muscular system of various species (e.g. [13,16,27]) are almost unusable for examinations regarding movement physiology. As an attempt to make at least biting physiology more accessible, here we provide muscle forces and muscle stress values of the mandible closer muscle of the general feeder *P*. *americana*, and compare these values with those of other species. Moreover, one of the best available bodies of literature regarding insect muscle physiology refers to cockroach species (e.g. [25,28]), which allows to compare our results with a variety of leg muscles with different task specific properties.

## Materials and Methods

### Animals

The cockroaches were kept at room temperature (23–25°C) and a humidity of 74% in perspex cages and fed twice a week with porridge oats; water was provided *ad libitum*. The laboratory colony used herein exists since 2012 and was originally derived from professionally bred animals (www.schaben-spinnen.de).

### Morphology and definitions

The blade-like mandibles (md, Fig 1) are connected via sail-like apodemes (mt, Fig 1) with the mandible closer muscles (m1, Fig 1); they originate from the baso-mesal edge of the mandibles and protrude into the head lumen (cp. [17]). The mandible opener (m2, Fig 1) attaches laterally at the base of the mandible. The left mandible has four distal teeth (1^l^-4^l^, Fig 1) and the right one three (1^r^-3^r^, Fig 1). Proximal to these teeth, a molar grinding area (mr, Fig 1) is present on both mandibles. Each mandible is connected to the head capsule via a hinge joint whose rotational axis is defined by an anterior (C_left_ and C_right,_ Fig 1A and 1B) and a posterior condyle. We defined the system of coordinates such that the horizontal plane was spanned by the joint axes of the left and right mandible joints with the connecting line between the two anterior condyles defining the horizontal axis of the head [see also 14]. The transverse plane was defined by these condyles and was always perpendicular to the horizontal plane. The sagittal plane, in turn, subtends the horizontal plane along the centreline between the anterior condyles and is perpendicular to both, the horizontal and the transverse plane (cp. Fig 1A in [14]). The mandibular axis was the line between the tip of the distal most tooth and the fulcrum of the mandible. The opening angle of the mandible (α, Fig 1A and 1B) was the angle between the horizontal axis of the head and the mandibular axis.

### Experimental setup

For the experiments ten adult specimens of the cockroach *P*. *americana* were used. 300 bites (250 of right mandibles) of eight animals (body mass given as mean ± s.d.: 1.12 g ± 0.17 g) with mandible opening angles covering the whole physiological range were analysed. The lower number of analysed animals resulted from accidental losses of distal teeth, which sometimes broke away during intense biting. As this study focuses on voluntary behaviour, a great quantity of bites was necessary to ensure that a sufficient number of near maximum forces was examined. Since our main interest was to provide a general view of the abilities and limitations of the cockroaches’ bite apparatus, we did not discriminate between individuals. According to expectable high inter-individual variations in muscle properties [29], as well as differences in bite motivation and muscle activation generally inherent in voluntary activity, we lumped all the single measurements together. This way we were able to determine the upper bite force limit over the whole range of opening angles. After measuring the voluntary biting forces, specimens were sacrificed by freezing (-18°C) for several minutes. Afterwards the forces necessary to open the mandibles of the dead animals were measured, i.e. the passive forces counteracting mandible opening. In preparation for the experiments, the animals were weighed with a laboratory scale (ABS 80–4, Kern & Sohn, Germany) and sedated by putting them in a refrigerator at 5°C for 10 minutes. They were then fixed supine onto an L-shaped aluminium holder, which was mounted via a lockable ball and socket joint onto a manual micromanipulator (Merzhäuser Wetzlar). Another small aluminium plate was inserted into two furrows of the holder such that a notch clasped closely around the throat of the cockroaches (Fig 1C). In this way, the animals were securely fixed and perturbations by the walking legs were prevented. Scale paper was mounted onto this aluminium plate, allowing for calibration of the video sequences used for kinematic analyses (see below). Dental cement (Harvard Cement, Harvard Dental International GmbH, Hoppegarten, Germany) was used to glue the cockroach heads into the corner of the L-shape such that the heads were perpendicular to the holder base. In order to see the mandibular joints during the biting process, the labrum was folded up and was also embedded in the dental cement. Dental cement was used here as it held the heads securely during the experiments but could be completely removed afterwards, and did not hamper subsequent μCT measurements [14]. Each specimen was treated for about one hour with synchronous measurements of kinematics and dynamics of the cockroaches’ biting behaviour. Consecutive measuring intervals within this period lasted 60 seconds each and were always followed by a pause of several minutes while the data was transferred, allowing the cockroaches some rest. Their mandibles were passively spread apart several times during these intervals by using the drives of the micromanipulator. For each specimen, the opening angles were randomly increased and decreased. If they did not voluntarily start to chew against the sensor tip the animals were spurred by air puffs or by touching their cerci with a fine brush or spring steel tweezers. Bites occurred typically in phases with frequencies of about 0.64 ± 0.12 s^-1^, whereas strong bites were rare, mostly occurring less than twice per 60 second measuring interval.

Alongside with active muscle contractions, passive elastic structures in the mandible joints or in the closer muscles can contribute to the closing movement if these structures are pre-stretched by the opener muscle or in the course of experimental manipulation. This way, the necessary power output of the closer muscles can be reduced. However, resting forces of the closer muscles, induced by minimal resting activity, may also contribute to non-zero forces between adjacent maxima. To assess possible muscular activity during the periods of rest, residual forces and corresponding opening angles were measured at these inactive intervals and compared to the passive forces measured after the cockroaches were sacrificed. Residual forces were determined for the instances directly prior to a contraction and are consequently restricted to medium and large opening angles.

During the experiment, right and left mandibles were measured. The order of the sides of the mandibles and the sequence of mandible opening angles depended largely on the cooperation of the particular specimen. Mostly, the examination started at the right mandible. Since the behaviour of the cockroaches was effectively unpredictable both side and opening angles were changed largely in random order. Since the mandibles are slightly asymmetric, the distances of the teeth to the joint axis differ between right and left mandibles. To take this characteristic into account, we measured the forces of both mandibles independently and calculated side-dependent muscle stress afterwards (see below). Since cockroach mandibles are comparably small, independent measurements of single mandibles allowed us to examine smaller and larger mandible gape angles than if the animals had bitten with both mandibles onto a force sensor placed between them. Moreover, only independent measurements of single mandibles can allow for clear distinction between the force contributions of the two appendages.

Since the closing movements of the two mandibles are largely coupled [30,31] fixation of one mandible might have a negative impact on the force measurement of the other one and was, thus, avoided. Single-sided mandible resection was excluded, too, as it led to heavy bleeding and the fast-clotting hemolyph can hamper the measurements considerably. Since it was not possible to prevent movements of the other (not measured) mandible completely both mandibles could interfere at small opening angles, just like during normal chewing (see results section).

### Kinematics

Kinematics were obtained with a Fastcam SA3 video system (Photron, San Diego, USA). The video sequences were captured with a frame rate of 50 Hz and lasted 60 s each. During the measurements the camera was positioned above the cockroaches with an angle of vision of 45° (Fig 1B and 1C). The distance between the lenses and the cockroach heads was about 0.2 m. This resulted in a resolution of 29 pixels per mm in the horizontal and 21 pixel per mm in the vertical dimension, due to perspective bias.

In order to calculate the mandible angle with respect to the horizontal line we digitized both anterior condyles of the mandible joints and the tip of the distal tooth of the respective mandible with the software package WINanalyze 3D (Version 2.1.1, Mikromak, Berlin, Germany). Since the labrum was folded up (Fig 1B), the heavily sclerotized condyles were clearly visible as dark marks in a matrix of lighter cuticle.

### Force measurements

**Fig 2:**
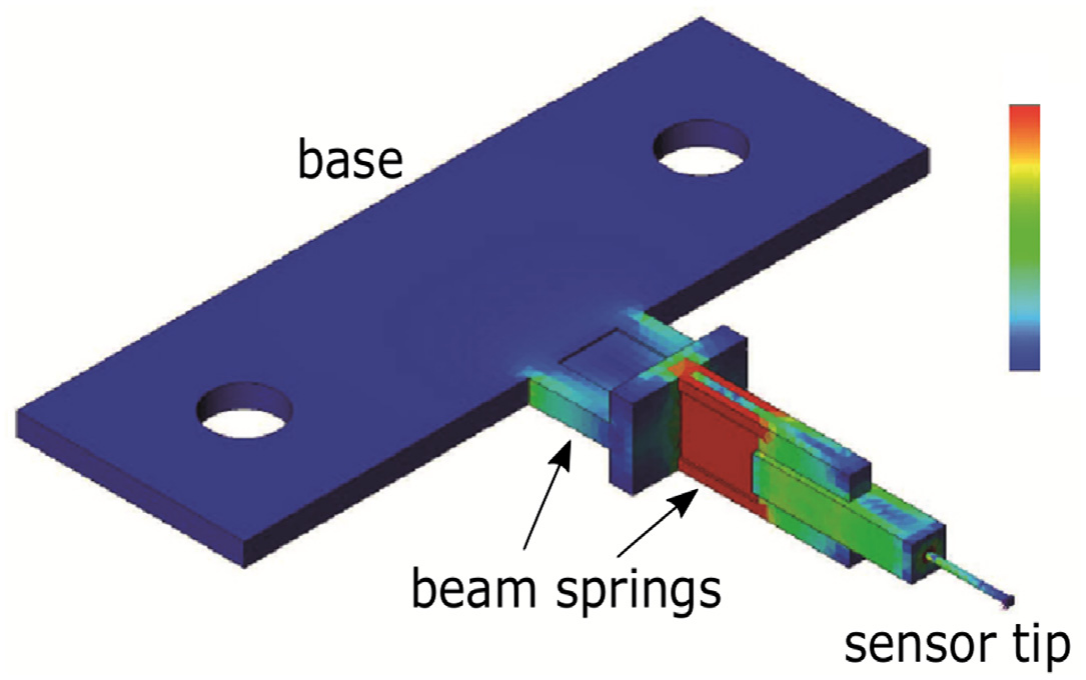
Finite element model of the force sensor’s general structure loaded laterally (Colours encode deformation of the structure; blue: no deformation and red: high deformation). The structural basis of the force transducer consisted of stereolithographically hardened PVC and provided two cantilever beam springs aligned perpendicular to each other; this way allowing for spatial resolution in two directions. The cross talk was very low in all loading situations. The sensitive structure was equipped with two semiconductor strain gages and integrated in a CNC-fabricated aluminium housing (see Fig 1c). For simplicity the bevelled sensor tip, i.e. the tip of the hypodermic needle that engaged with the mandible teeth was modelled as a cylinder.

**Fig 3:**
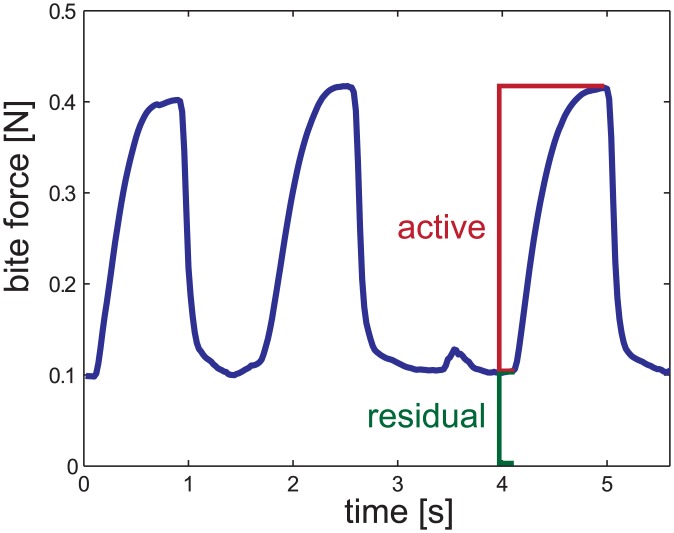
Example sequence of measured *F*_*tot*_. The residual forces *F*_*res*_ changed little during a trial and are generated by slow muscle fibres and passive elasticity of the muscle-joint complex while the changes on top of this basis (*F*_*act*_) are generated by faster muscle fibres.

**Fig 4:**
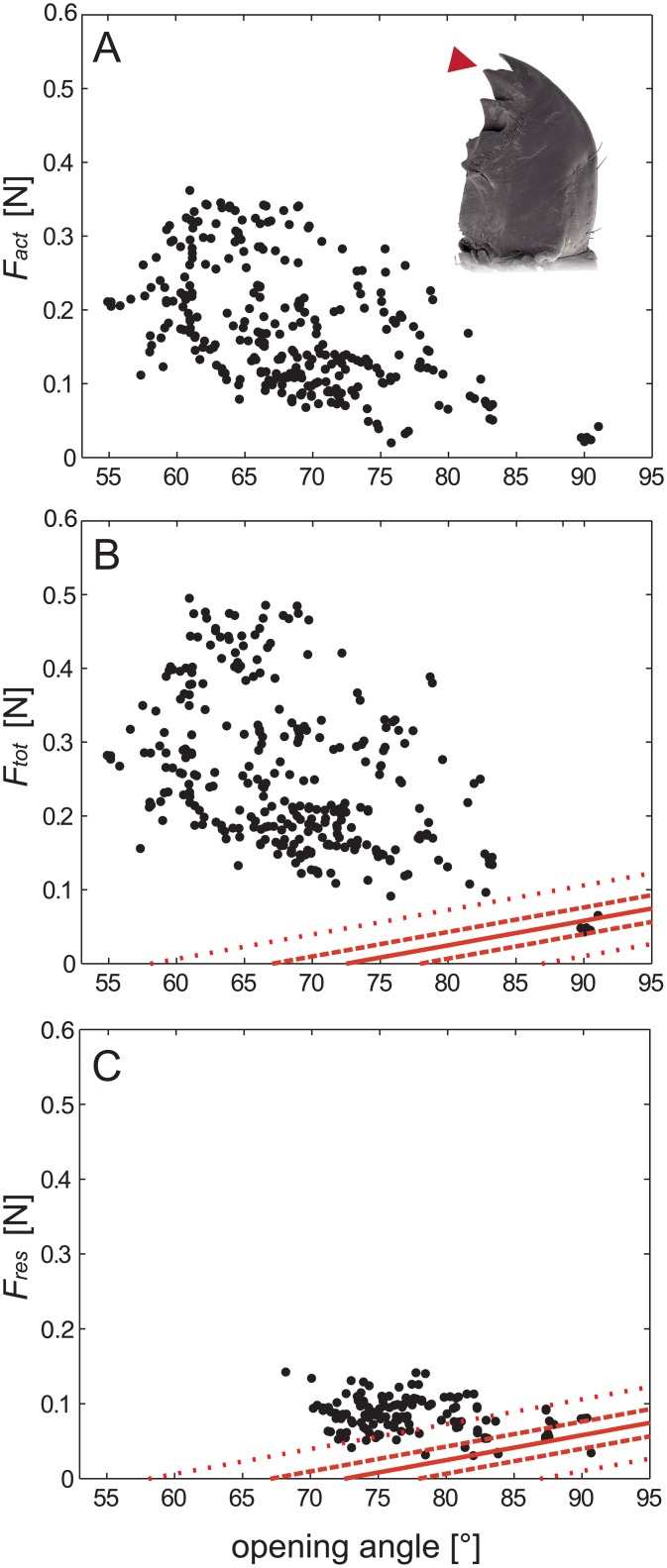
Resulting forces at the position of the second right and third left teeth over the functional range of the mandibles. A) *F*_*act*_, i.e. forces generated by the faster muscle fibres (for details see text). B) *F*_*tot*_, i.e. total bite forces including tonic and passive contributions. C) Residual forces at the position of the second right and third left teeth over the functional range of the mandibles. Residual forces represent the sum of passive joint forces and tonic contributions while their effective contributions depend on the opening angle. Forces were determined for the instances directly prior to a contraction and are consequently restricted to medium and large opening angles. In B and C, the red lines show the mean (solid), ± standard error (s.e.m.; dashed) and ± standard deviation (s.d.; dotted) of the passive forces, i.e. those forces necessary to open the mandibles of freshly killed specimens. At small and medium opening angles, the residual forces (*F*_*res*_) are primarily generated by tonic muscle fibres while at high opening angles passive forces dominate.

A self-built 2D force transducer was used for the measurement of the bite forces (Fig 2). Camera and transducer were triggered synchronously. The structural basis of the force transducer was built layer-by-layer by stereolithographically hardened PVC and consisted of two cantilever beam springs aligned perpendicular to each other (Fig 2). The beam for measuring the horizontal forces was placed on top of the unit for the vertical force components, with material thicknesses resulting in equal stiffnesses for both components. When loaded with 0.5 N the tip of the sensor was deflected by about 0.5 mm, which corresponds to an angular change of the mandible position of about 10°. However, particularly during the measurement of large opening angles the sensor was significantly preloaded by passive and tonic forces (see results, Figs 3 and 4) which led to reduced sensor deflections.

Natural frequencies of both directions exceeded 1 kHz. The sensor provided sensitivities of 67 mV/N in the horizontal, and 54 mV/N in the vertical direction. Cross talk was negligible (less than 1%). To optimize the very tip of the sensor for fitting between the teeth of the mandibles without affecting the stiffness negatively, a 10 mm long piece of a hypodermic needle with a diameter of 0.8 mm was fitted in a preformed hole such that the bevelled tip protruded about 5 mm from the sensing structure. The transducer unit was installed in a CNC-fabricated aluminium housing and wired with a shielded low-noise cable. We used the MGCplus system (Hottinger Baldwin Messtechnik, Darmstadt, Germany) for data acquisition and pre-processing. The amplifier module ML10B with the AP01i connection board was used to integrate the semiconductor strain gages into Wheatstone bridge circuits with 1 V bridge excitation. The sample rate was 200 Hz and a 20 Hz Bessel low pass filter was chosen for signal smoothing. We set the nominal value to 150 mV/V for amplifying the signals. A desktop PC with the data acquisition software Catman Easy V3.3.3 (Hottinger Baldwin Messtechnik, Darmstadt, Germany) was connected by an USB communication processor (CP22). During subsequent analyses only every 4th data point was taken into account such that kinematics and forces had the same sample frequency. Calibration was carried out analogous to the more complex procedure described in Reinhardt and Blickhan [32] for a 3D sensor.

During the experiments, the bevelled tip of the hypodermic needle was always inserted horizontally in the groove between the first (1l, 1r, Fig 1) and second (2l, 2r, Fig 1) teeth of the mandible. At curved structures, such as claws and toothed jaws, the direction of forces acting on protruding objects can deviate significantly from the perpendicular line to their main axes [33–35]. The crucial measure is the total force acting on a food item, however 1D force transducers cannot account for variable force directions. Thus, by using a 2D transducer we could measure the dorso-ventral and horizontal dimensions of the bite forces separately and calculate the resulting force from these perpendicular forces afterwards. The bite forces were read out only for the smallest opening angle of a specific biting event. At this point the force values were maximal and contraction speeds of the closer muscles were zero, i.e. all bite force measurements referred from here onwards were considered as isometric. Thereby positive horizontal forces were medio-lateral forces pointing into the sagittal plane of the animal and positive dorso-ventral forces point dorsally out of the horizontal plane, i.e. the mandible pulled on the transducer tip. Additionally, the force traces were used for the determination of the duration of the bite events. The starting point was defined as the instant when the resulting force rose above the resting force, and the bite event ended when the force fell back to initial values (Fig 3 and S1 Fig).

At smaller opening angles, below about 60°, the contralateral mandibles could interfere with each other (Fig 1a). Such interactions were either kinematically visible or were revealed by odd forces traces. If one of these artefacts occurred bites were excluded from the analyses. The vertical position of the sensor tip was not readjusted after initial alignment and the mandibles have only one rotational degree of freedom. Thus, depending on the initial positioning, vertical resting forces applied due to geometric constraints could exceed 0.2 N for the highest opening angles. These induced forces were not considered as physiological relevant and accordingly were not included in the calculation of the bite forces.

### Data processing

All data were processed with MATLAB R2010a (The MathWorks, Natick, MA, USA). Significance was tested by using paired Student’s t-test.

Due to the elevated position of the camera (see above), the vertical dimension of the video sequences was generally biased and the vertical resolution was lower than in the horizontal direction. Moreover, the positioning of the cockroach heads with respect to the camera differed slightly between the specimens. To take these potential sources of error into account, the position of the distal mandible tip was corrected by using the real mandible length, as determined from μCT images [14]. The horizontal position was taken from the video sequences, while the vertical position was recalculated according to the Pythagorean theorem: the horizontal position being the leg of the triangle adjacent to the opening angle of the mandible, and the real length of the mandible being the length of the hypotenuse. The opening angle was calculated subsequently as the angle between the horizontal line as defined above and the mandible axis (cp. Fig 1A and 1B).

Active horizontal and vertical forces were measured directly as the difference between the initial residual force and the peak force of the current bite. For the calculation of the effective external bite forces, horizontal residual forces were considered but the vertical resting forces were not, as these were imposed by the experimental setup (see above). The vector sum of these measures was the resulting active force ($F_{act}=\sqrt{F_{hor}{}^{2}+F_{vert}{}^{2}}$) or the resulting total force ($F_{tot}=\sqrt{{\left(F_{hor}+F_{res}\right)}^{2}+F_{vert}{}^{2}}$), i.e. the total force acting at the transducer tip, with *F*_*hor*_ being the horizontal force component, *F*_*vert*_ the vertical force component and *F*_*res*_ being the residual force in horizontal direction. *F*_*res*_ consisted of forces generated by the resting tonus of the muscles and the passive closing forces caused by the stiffness of the joint and adjunct membranes. These components were resolved afterwards by determining the passive contributions of the joint structures themselves (see below). External forces were calculated and normalized to the second right and third left teeth, which are equally distant to the attachment points of the tendons and probably provide the major biting site. The ratios of the distances of the teeth to the joint axis determine the bite forces available. Thus, at the distal-most teeth available forces are lower. The distances of these teeth to the joint axes were about 2.52 mm in both mandibles, and did not differ significantly from each other [14]. Accordingly, resulting forces were about 8% lower than the forces at the second right and third left teeth, whose distances to the joint axes were about 2.32 mm.

Taking account of the individual effective mechanical advantages of the mandibles muscular forces could be determined. According to the anatomical values documented in [14], and the distances between the mandible joints and the positions of the second right and third left teeth (see above), the mechanical advantage of the two mandibles was about 0.4. It was calculated as the quotient of the length of the effective input lever and the length of the external lever. The effective input lever corresponds to the projection of the anatomical input lever, i.e. the connecting line between the mandible joint axis and the attachment point of the mandible closer apodeme, projected onto the horizontal line (cp. Fig 1A). In closed mandibles, the angle between both inner levers and the horizontal line is about 0° and increases with increasing mandible opening. Consequently, the effective input lever shortens according to the cosine of the included angle. Using equilibrium of moments, internal muscle forces *F*_*m*,*act*_ or *F*_*m*,*tot*_ were calculated from external forces *F*_*act*_ or *F*_*tot*_, respectively. Mean passive joint forces (see Figs 4B and 1C) were subtracted from the residual forces prior to the calculation of *F*_*m*,*tot*_.

The cross-section area of the mandible closer muscles was taken as the sum of the cross-section areas of the eight muscle bundles, each comprising several dozens of muscle fibres that make up each muscle. The areas of the single fibre bundles, in turn, were determined in the software package Amira 5.2.2 (Visage Imaging Richmond, Australia) by measuring virtual cross sections perpendicular to the muscle fibre directions as obtained by μCT measurements (for details see [14]).

## Results

The examined mandible movements occurred in an angular range from almost closed to maximally open mandibles. When mandibles are closed the opening angle is about 47° ± 1° in the right and 49° ± 1° in the left mandible [14]. By examining voluntary movements of the untreated mandibles maximum voluntary opening angles up to 103° could be observed, with 3 of 8 specimens opening their mandibles to more than 99°. During the force measurements the minimum opening angle was limited by the inserted sensor tip to about 55°. Bite durations ranged from 0.24 s to 1.52 s whereas strong bites with *F*_*tot*_ > 0.34 N lasted considerably longer (0.49 s ± 0.13 s vs. 0.99 s ± 0.21 s). Since the contributions of the different individuals were uneven only four specimens could be used for statistical testing of the durations of strong and weak bites. For these individuals ten strong bites with total forces above 0.28N (0.32 N ± 0.07 N) and ten weaker bites (mean: 0.17 N ± 0.024 N) were sampled, merged respectively and then tested against each other. The paired t-test yielded highly significant differences (p<0.001) between the bite durations of strong (0.68 s ± 0.26 s) and weaker bites (0.45 s ± 0.11 s).

**Fig 5:**
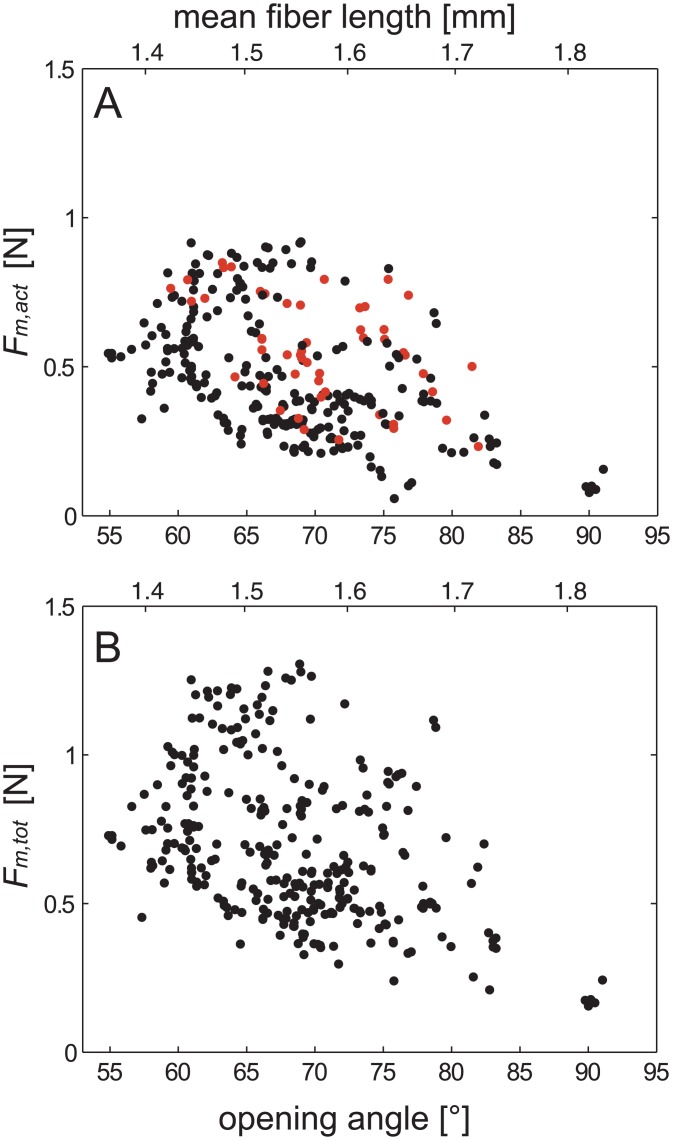
Contraction forces of the mandible closer muscle over the functional range of the mandibles in degrees (lower abscissa) and the mean fibre length of the closer muscle in mm (upper abscissa). A)*F*_*m*,*act*_ i.e. forces generated by faster muscle fibres. The values for left (red) and right mandibles are highly concordant B)*F*_*m*,*tot*_. i.e. total muscular forces. Mean passive forces (see Figs 4b and 5) were subtracted from the residual forces prior to the calculation of *F*_*m*,*tot*_. Mean muscle fibre lengths and length changes were described in [14].

Active bite forces (*F*_*act*_) were measured in the range from 55° to 91°. They changed with the opening angles of the mandibles and attained maximum values of 0.36 N at opening angles of about 61° (Fig 4A). According to the mechanical advantages of the mandibles, the forces generated by the closer muscles (*F*_*m*,*act*_) were calculated. Maximum values of about 0.92 N were attained at a force plateau between about 60° and 75° (Fig 5A). With an effective cross section area of about 2.23 mm^2^ [14] a maximum muscle stress of 41 N/cm^2^ is found. At opening angles above about 85° *F*_*act*_ values almost disappear, and were smaller than passive forces. Maximum values and angular distributions were very similar for left and right mandibles (Fig 5A).

External residual forces arise from muscular resting tension, real passive forces exerted by elastic joint structures, and elasticity of the muscles themselves. They were measured for an angular range from 68° to 94° in living (n = 142) and between 63° to 117° for dead specimens (n = 37). Real passive forces, as measured in the sacrificed animals, increased with rising opening angles for each specimen. On average, noticeable passive forces occur only at opening angles above 70° and reach considerable values at angles exceeding 85°, i.e. at high opening angles (Fig 4B and 4C). At maximum voluntary opening angles of about 100°, passive forces are about 0.09N. However, though highly variable, residual forces in living animals were also considerable at lower opening angles of 70°—75° (Fig 4C). Hence, passive joint forces provide only a small part of these residual forces. Residual forces have to be generated by muscle activity instead, and are expected to rely on persistent tension in specific slow muscle fibres (see discussion). Thus, if assuming the residual forces as intrinsic muscle forces they add up with the active muscle forces (*F*_*act*_) to *F*_*tot*_, i.e. the total forces acting at the mandible edge. *F*_*tot*_ is markedly higher than *F*_*act*_ and reaches maximum values of about 0.5 N (Fig 4B). However, the total forces also decline considerably at higher opening angles. If calculating the forces generated by the mandible closer muscle based on *F*_*tot*_, maximum values of up to 1.3 N result within an angular range from 60° to about 75° (Fig 5B). These maximum forces would result in muscle stress values of about 58 N/cm^2^. Left mandibles were not tested as many times as those on the right. Since residual tension depends on previous activity (see discussion) residual forces were considerably lower in the left mandibles than in right mandibles (0.062 N ± 0.041 N vs. 0.1 N ± 0.042 N) and the maximum values of *F*_*tot*_ accordingly. However, only in two specimens both, left and right bites were analysed, which prevented reliable statistical testing. Therefore we tested the influence of previous bite activity on residual forces by comparing residual force values of the initial trial against those of one of the last trails of the same specimen. To this end ten force values were sampled for each trial (first and last) over the whole angular range of the mandibles in all examined specimens. These values were merged respectively and tested subsequently. The paired t-test yielded highly significant differences (p<0.001) between the residual forces at the beginning of the treatment (0.071 N ± 0.033 N) and those after sustained activity (0.125 N ± 0.042 N).

## Discussion

Mandibles are the strongest mouthparts in insects with biting mouthparts, including cockroaches. They perform a wide range of tasks, from very gentle manipulations to those where maximum bite forces are needed. Since *P*. *americana* are opportunistic feeders, their food spectrum is particularly wide. With their sharp mandible teeth, they even feed on tough materials such as wood and leather [36]. Thus, the 2^nd^ right (2^r^, Fig 1) and 3^rd^ left (3^l^, Fig 1) teeth seem to form an analogous structure to carnivoran carnassials. Like in this well-known structure, a pair of sharply edged teeth move closely past each other, allowing them to shear tough fibrous materials (cp. [37]). In this way, functional requirements of the cockroach’s chewing apparatus are more similar to those of ground beetles than those of stag beetles, which may explain why the muscle stress values are quite similar to the former and rather different from the latter (see below).

### Forces

The upper edge of *F*_*m*,*act*_ plotted against the opening angle resembles a typical force-length relation as found in many skeletal muscles (e.g. [38,39]). It is characterised by an ascending limb at low opening angles, a force plateau at intermediate, and a descending limb at high angles. This holds also true when *F*_*m*,*act*_ is plotted against the mean fibre lengths (Fig 5A). Though values are generally higher in *F*_*m*,*tot*_ it shows a similar angular dependency. Particularly strong bites, i.e. those data points shaping the upper edge of the scatter diagram, lasted markedly longer than the weaker bites (see results section). This indicates that the muscle fibres require sufficient time to reach their maximum forces and provides evidence for full voluntary excitation. In contrast to the external forces (*F*_*tot*_), passive forces were excluded from the calculation of *F*_*m*,*tot*_ (see above). Therefore, the force differences to *F*_*m*,*act*_ necessarily have to be provided by muscular structures. While *F*_*m*,*act*_ seems to be generated by relatively fast muscle fibres, this second muscle driven force component occurred almost continuously during a trial. Hoyle [40] examined very similar residual forces in jumping muscles of some orthopteran species and Chesler and Fourtner [41] found it even in metacoxal muscles of *P*. *americana*; they called it basic tonus or residual tension, respectively. They showed that specialized slow muscle fibres are responsible for generating these forces, and found that the residual tension can require several minutes for complete relaxation, even if no further activation was supplied. However, tonic forces rely on muscle activity and are therefore subject to muscle length changes. Consequently, they decrease at high opening angles (cp. Fig 4C). Eventually, at mandibular opening angles above 85°, the passive forces exceed both active and tonic muscle forces, and are the main drive of mandible closure. Moreover, tonic forces seemingly depend on the extent of prior muscle activity (cp. [42]). Thus, residual forces were significantly higher after sustained activity of the mandibles (see results). They were also considerably higher in the more extensively tested right mandibles (n = 250) than in those on the left (n = 50). Accordingly, Ache and Matheson [42] demonstrated, for the muscles driving the femur-tibia joints of the hind legs in the locust *Schistocerca gregaria*, that the extend of prior muscle activity determines subsequent resting tonus, at least as long as the activity of corresponding fibres were not inhibited by common inhibitory motor neurons. Since elastic properties of the substrate significantly affect maximum bite performance and fibre recruitment in a wide range of species [43] the occurrence of strong tonic forces in our experiments is likely to be the result of the elastic properties of the force sensor and of the experimental procedure. Nevertheless, tonic muscle tension seems to have particular significance when the animals chew on tough or resilient materials without the necessity of active mandible opening. When this occurs, tonic forces add to the forces generated by faster muscle fibres and provide distinctly enhanced bite forces. During quick actions, in turn, the slow tonic fibres could not activate fast enough to increase bite forces significantly. When fast mandible opening is required, active slow fibres, with their typically high latencies, may even hamper the release and subsequent grasping, particularly since the mandible openers have only about one-tenth of the cross section area of the closer muscles and act via much shorter levers (Fig 1A). Therefore, tonic fibres should be activated only during sustained grasping or chewing behaviour. Under these circumstances, however, the employment of slow muscle fibres provides a tool for the generation or supplementation of very efficiently generated muscle forces [44,45] with only a minimum of cross section area, and therefore head volume, required.

### Comparative considerations

In *P*. *americana*, maximum muscle stress varies from about 41 N/cm^2^ to 58 N/cm^2^ depending on the contribution of tonic forces. Similar values were found by Wheater and Evans [7] in thirteen ground and rove beetle species. In their study the values ranged from 31 N/cm^2^ to 56 N/cm^2^ (46.5 ± 6.8 N/cm^2^) while the species *Pterostichus madidus* showed the highest mandibular closer muscle stresses. Even higher muscle stress values were found in the chelae closer muscles of several crustacean species and in the chelicerae of a solifugae species [21,22]. In all of these cases, the high stress values of up to 100 N/cm^2^ coincide with specific behaviours such as feeding on hard shelled prey or digging. Further muscle stress related comparisons, considerations regarding the muscle stress requirements of leaf-cutting ants and the relevance of different mandible opening angles in a variety of species are addressed in the supporting information (S1 Text).

The differences between the maximum muscle stresses of the mandible closer muscles in stag beetles (*Cyclommatus metallifer*) and cockroaches are considerable, while those of ground beetles are largely similar to that of the less related cockroach; the muscle stress values for stag beetles are about 18 N/cm^2^ [6] whereas those of ground beetles and cockroaches are more than twice as high. These findings seem to reflect the main purpose of the appendages: while ground and rove beetles, and cockroaches, use their mandibles for food acquisition and shredding, stag beetles feed on plant sap and males use their enormous mandibles almost exclusively for conspecific fighting. Females, in turn, use their mandibles to dig into rotten wood or soft soil for oviposition [6]. The most determining requirement, however, seems to be the males’ fight over females. Thus, stag beetles, though able to generate high forces with their mandibles, use them primarily to clutch on their opponents, requiring comparatively smaller forces than shredding. Moreover, the larger males have particularly broadened heads, providing extensive attachment areas and volume for the mandible muscles. In this way, the required force values can be obtained by this increase of the effective cross section area. However, the ability to quickly seize the rival seems to be an important feature of their highly specialised mandibles. This is indicated also by the small mechanical advantage of about 0.13 to 0.28, depending on the grip position (cp. [6]), resulting in high velocity gear ratios. Thus, by exploiting the available space and attachment area within these beetles’ heads, relatively fast but weak muscle fibres can generate the required forces and also fulfil the demand for fast muscle contractions.

The consequently enlarged heads and the big mandibles hamper all the more the locomotor agility of stag beetles by shifting their centre of mass anteriorly, which increases the body’s moment of inertia (cp. [46]) and may have negative effects on locomotor stability [10,47]. Having relatively smaller muscles, in turn, even if at the cost of slower contractions allows smaller insects a better balanced body shape and higher agility along with high bite forces. Thus, in contrast to the generally more agile ground beetles and cockroaches, the larger head size and, at least in male stag beetles, required fast mandibular actions seem to cause the significant differences in the muscle stress values.

## Conclusion

In terrestrial arthropods, largely increased body size creates problems with the moulting process and breathing [48–50], consequently growth is limited. In general, terrestrial arthropods occupy mostly ecological niches which are characterised by small body sizes. The evolutionary trend towards high muscle stress, i.e. long sarcomeres in turn, provides the ability for generating high forces with relatively small cross section areas and limited space requirements. This solution that seems optimal for small animals with exoskeletons appears to be specific for arthropods, and is not implemented equivalently in vertebrates with their typical endoskeletons. Most vertebrates are not restricted by external shells, hence, if necessary, they can increase the cross section areas of stressed muscles much more easily. However, in the cockroaches’ chewing apparatus, the employment of an additional second force generating mechanism as found in tonic fibres provides the ability to fulfil both major requirements, fast movements and high biting forces with relatively small muscles in a small head capsule. Hence, they join the properties of the specialized slow crusher and faster cutter chelae of many crabs and crayfish [51–53] into one device.

## Supporting Information

S1 Fig Example force trace showing the bite durations of weak, strong and intermediate bites. Strong bites lasted generally longer than weaker ones (see results section).
(EPS)

S2 Fig Resulting forces at the position of the second right and third left teeth over the functional range of the mandibles. In contrast to Fig 4A the contributions of single specimens are resolved and colour coded. The legend assigns individual specimens (numbers) and corresponding colours.
(TIF)

S1 File Dataset providing all data points of the Figs 4A,4B and 5. All abbreviations used in the header of the file comply with the abbreviations in the manuscript. Thus ‘opening angle’ refers to the opening angle of the mandibles, ‘Fact’ is *F*_*act*_, ‘Ftot’: *F*_*m*,*tot*_, ‘Fm,act’: *F*_*m*,*act*_, Fm,tot: *F*_*m*,*tot*_, ‘specimen number’ identifies the examined specimen and ‘side’ indicates whether a right or a left mandible was examined.
(TXT)

S2 File Dataset providing the data points of Fig 4C. All abbreviations used in the header of the file comply with the abbreviations in the manuscript. Thus ‘opening angle’ refers to the opening angle of the mandibles, ‘Fres’ is *F*_*res*_, ‘specimen number’ identifies the examined specimen and ‘side’ indicates whether a right or a left mandible was examined.
(TXT)

S3 File Dataset of the passive forces caused by joint resistance as shown in Fig 4B and 4C. The terms in the header refer to terms used throughout the manuscript. Thus ‘opening angle’ refers to the opening angle of the mandibles, ‘passive forces’ refers to those forces necessary to open the mandibles of freshly killed specimens, ‘specimen number’ identifies the examined specimen and ‘side’ indicates whether a right or a left mandible was examined.
(TXT)

S1 Text Comparisons with muscle stress data from the literature, muscle stress requirements of leaf-cutting ants and the relevance of different mandible opening angles in a variety of species. (PDF)
