# Supplementary figures and images for: Fast and Powerful: Biomechanics and Bite Forces of the Mandibles in the American Cockroach Periplaneta americana
Source: PLoS One. 2015 Nov 11;10(11):e0141226. doi: 10.1371/journal.pone.0141226 (PMC4641686; doi:10.1371/journal.pone.0141226)

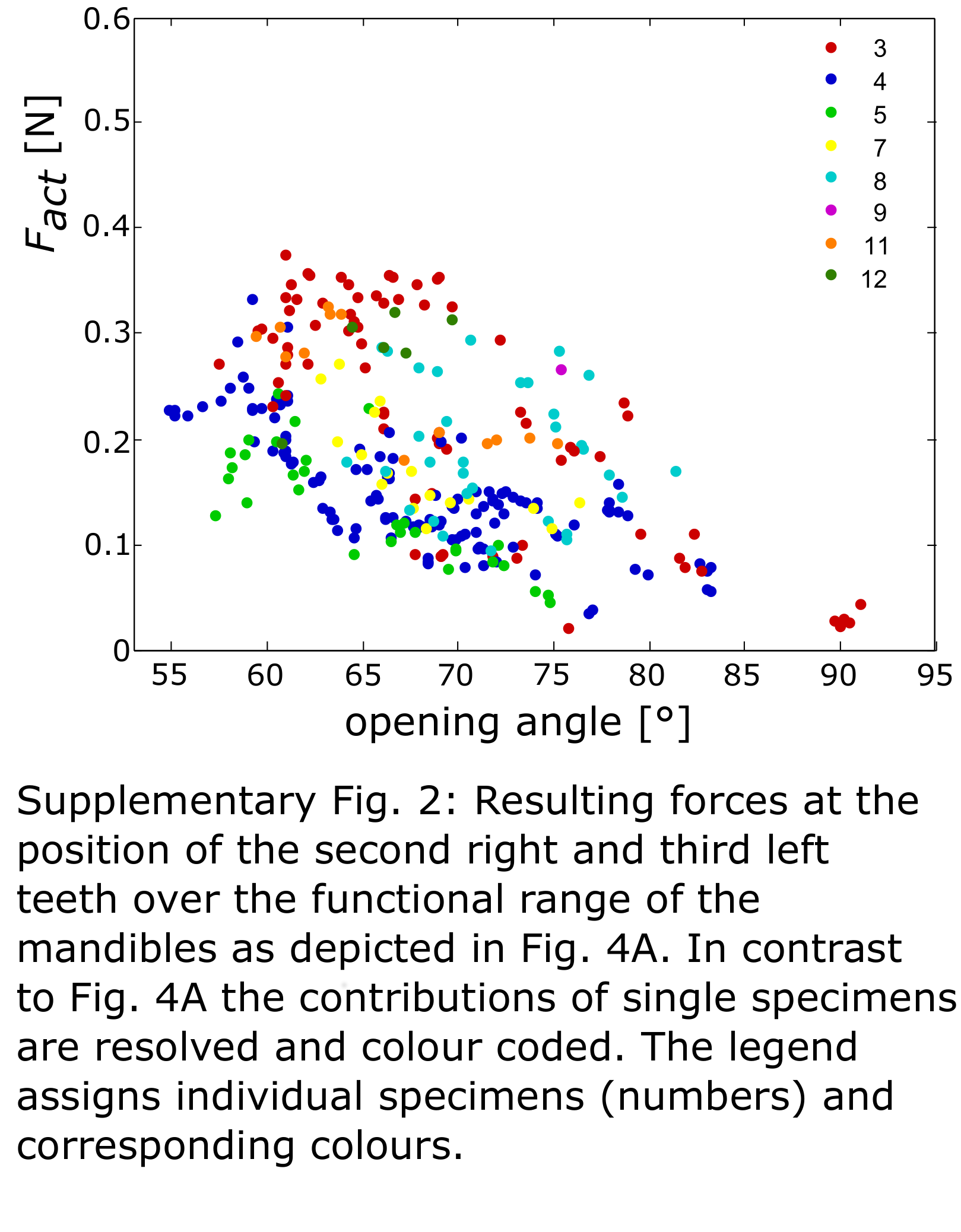

Supplement: S2 Fig — In contrast to Fig 4A the contributions of single specimens are resolved and colour coded. The legend assigns individual specimens (numbers) and corresponding colours. (TIF) [file pone.0141226.s002.tif]
