# Supplementary material for: Fast and Powerful: Biomechanics and Bite Forces of the Mandibles in the American Cockroach Periplaneta americana
Source: PLoS One. 2015 Nov 11;10(11):e0141226. doi: 10.1371/journal.pone.0141226 (PMC4641686; doi:10.1371/journal.pone.0141226)
Supplement: S1 Text — (PDF) [file pone.0141226.s006.pdf]

S1 Text. Comparisons with muscle stress data from the literature and muscle stress requirements of leaf-cutting ants

In *P. americana* we found maximum muscle stress of mandible closer muscles in the range of 41 N/cm^2 to 58 N/cm^2. Relatively high muscle stresses were also found in a leg muscle of *P. americana* which is involved in the positional control of the hips [1]. This muscle (179) may generate a tetanic muscle stress of up to 47 N/cm^2. Since our experiments rely on voluntary behaviour, we can only speculate on the extent of mandible closer excitation on the basis of bite durations and forces (see the main manuscript). Nevertheless, cockroach muscle 179 shows much lower muscle stresses when stimulated with *in vivo* stimulation patterns, reaching only 20 N/cm^2 in this case.

To our knowledge, except for the males of stag beetles whose mandibles are highly derived weapons utilized in male–male fights for mating opportunities and are no longer used for food processing [2], only one other study deals with biting forces over a wider angular range. In the claws of the examined crab *Scylla olivacea* [3] maximum bite forces occurred at intermediate opening angles whereas, in contrast to the mandibles of *P. americana*, the dactyl and the propodus did not slide against each other. The larger gape seems to reflect the mean size of the crabs' preferred food, i.e. the mean shell diameter of their preferred mussel prey [4]. Maximum bite forces of *P. americana* occur just before the teeth of the contralateral mandibles begin to interdigitate (cp. Figs. 4 and 1A). Thus, the most robust structures cockroaches are faced with seem to be small, thin or fibrous, such as plant fibres or exoskeletons of dead arthropods. The relatively small molar regions (mr, Fig. 1A) of the mandibles, responsible for grinding up shredded material, engage at even smaller opening angles. The limited bite force available at these angles (Fig. 4), seems to be compensated by the more proximal position of the molar regions, their shorter out-levers and consequently higher mechanical advantage. In male stag beetles, however, bite force seems to increase continuously with decreasing muscle fibre length. Particularly if the opponent starts to slip off, the increasing grip forces here may prevent an escape and increase the beetle's fitness to fight.

Leaf-cutting ants belong to the strongest biting insects. Some species are specialized in cutting hard leaves and even grass stalks [5]. The dimensions of their mandibles, including the closer muscles, are well-investigated in some species [6,7]. Moreover, also some data regarding the sarcomere lengths are available, which are about 5-9 µm [8]. Such long sarcomeres imply high muscle stresses, as the number of independent force generators (myosin heads) acting in parallel increases with the length of the myosin filament. When applying our results (maximum stress of about 50 N/cm^2) to the mandible closer muscles of these ants, which have effective cross section areas of about 4 mm^2 in major workers [cp. 7], muscle forces of up to 2 N can be obtained. Since the mechanical advantage is similar to the mandibles of *P. americana* and even higher in the more proximal cusps, bite forces significantly exceeding 1 N can be expected. Schofield et al. [6] showed that unworn mandibles of leaf-cutting ants need only forces of around 0.15 N to cut vein-free regions of relatively tough leaves. However, cutting leaf-veins and cutting with worn mandibles requires much higher forces [6]; the forces required for cutting vein-free leaf regions with worn mandibles were about 0.35 N, which is still attainable if maximum muscle stress is similar to, or higher than, that found in cockroaches and ground beetles. By contrast, much lower stress values of about 18 N/cm^2, similar to those of stag beetles [2], would result in maximum bite forces of only 0.35 N which is certainly not sufficient to cut apart leaf veins.

1. Ahn AN, Full RJ (2002) A motor and a brake: two leg extensor muscles acting at the same joint manage energy differently in a running insect. J Exp Biol 205: 379-389.
2. Goyens J, Dirckx J, Dierick M, Van Hoorebeke L, Aerts P (2014) Biomechanical determinants of bite force dimorphism in *Cyclommatus metallifer* stag beetles. J Exp Biol 217: 1065-1071.

3. Yap NWL, Lin Y, Todd PA (2013) Chelae force generation at variable gape sizes in the mud crab, *Scylla olivacea* (Brachyura: Portunidae). Nature in Singapore 6: 179-185.
4. Hughes RN, Elner RW (1979) Tactics of a predator, *Carrcinus maenas* and morphological responses of the prey, *Nucella lapillus*. Journal of Animal Ecology 48: 65–78.
5. Moll K, Roces F, Federle W (2010) Foraging grass-cutting ants (*Atta vollenweideri*) maintain stability by balancing their loads with controlled head movements. J Comp Physiol A Neuroethol Sens Neural Behav Physiol 196: 471-480.
6. Schofield R, Emmett K, Niedbala J, Nesson M (2011) Leaf-cutter ants with worn mandibles cut half as fast, spend twice the energy, and tend to carry instead of cut. Behavioral Ecology and Sociobiology 65: 969-982.
7. Paul J, Gronenberg W (1999) Optimizing force and velocity: mandible muscle fibre attachments in ants. J Exp Biol 202 (Pt 7): 797-808.
8. Gronenberg W, Paul J, Just S, Holldobler B (1997) Mandible muscle fibers in ants: fast or powerful? Cell Tissue Res 289: 347-361.
